# The Protective Effect of 5-Aminosalicylic Acid against Non-Steroidal Anti-Inflammatory Drug-Induced Injury through Free Radical Scavenging in Small Intestinal Epithelial Cells

**DOI:** 10.3390/medicina56100515

**Published:** 2020-10-01

**Authors:** Eun Suk Jung, Hyun Joo Jang, Eun Mi Hong, Hye Li Lim, Sang Pyo Lee, Sea Hyub Kae, Jin Lee

**Affiliations:** Division of Gastroenterology, Department of Internal Medicine, Dongtan Sacred Heart Hospital, Hallym University School of Medicine, Hwaseong-si, Gyeonggi-do 18450, Korea; esjung@hallym.or.kr (E.S.J.); em1204@hanmail.net (E.M.H.); hyeli2676@gmail.com (H.L.L.); ultrapyo@hallym.or.kr (S.P.L.); kaeyoo@hallym.or.kr (S.H.K.); jinlee@hallym.or.kr (J.L.)

**Keywords:** 5-aminosalicylic acid, enteropathy, free radical scavenging, non-steroidal anti-inflammatory drug, small bowel injury

## Abstract

*Background and objectives:* Non-steroidal anti-inflammatory drugs (NSAIDs) have been among the major causes of small intestinal injury in clinical practice. As such, the current study investigated the protective effect of 5-aminosalicylic acid (5-ASA) against an NSAID-induced small intestinal injury. *Materials and Methods*: IEC-6 cells were treated with various concentrations of indomethacin with or without 5-ASA in a serum-free medium, after which an 3-(4,5-Dimethylthiazol-2-yl)-2,5-Diphenyltetrazolium Dromide (MTT) assay, a cell apoptosis assay, a caspase-3 activity assay, a reactive oxygen species (ROS) content and Superoxide dismutase 2 (SOD2) activity measurement, a Western blotting for occludin and zonula occludens-1 (ZO-1) and a wound healing assay were conducted. *Results:* 5-ASA ameliorated indomethacin-induced cell apoptosis and an increase in the intracellular ROS content while augmenting the indomethacin-induced suppression of SOD2 activity in IEC-6 cells. Moreover, 5-ASA reversed the indomethacin-induced attenuation of occludin and ZO-1 expression and promoted faster wound healing effects in IEC-6 cells following an indomethacin-induced injury. *Conclusions:* Our results suggested that 5-ASA protects small intestinal cells against an NSAID-induced small intestinal injury by scavenging free radicals. Therefore, 5-ASA could be a potential treatment for an NSAID-induced small intestinal injury.

## 1. Introduction

Non-steroidal anti-inflammatory drugs (NSAIDs) have been among the most commonly prescribed medications worldwide for several conditions including rheumatoid arthritis, osteoarthritis and musculoskeletal injuries [1]. However, NSAIDs have been shown to cause various gastrointestinal complications such as ulceration, bleeding, perforation, stricture and obstruction [2,3]. Although NSAID-induced gastrointestinal injuries have been mainly reported to occur in the upper gastrointestinal tracts, NSAID enteropathy has slowly become more prevalent than NSAID gastropathy [3]. Following advancements in techniques for small bowel evaluation including capsule endoscopies and double balloon enteroscopies, incidences of NSAID-induced small bowel injuries have hovered around 53–80% among healthy short-term users with an estimated prevalence of 50–71% among long-term (>3 months) daily users [4,5,6,7,8].

NSAIDs induce small bowel injury through mitochondrial damage, which leads to calcium efflux and free radical generation that subsequently break down the intercellular integrity and increase intestinal permeability [3]. Although several medical treatments have been attempted to manage or prevent an NSAID-induced enteropathy, their effects had been limited. Coadministration of proton pump inhibitors was not effective in preventing an NSAID-induced enteropathy. An animal study showed that proton pump inhibitors may exacerbate an NSAID-induced enteropathy by shifting the enteric microbial population [9]. Moreover, a small observational study revealed that misoprostol had an effect in the treatment of aspirin-induced enteropathy [10] while other studies have indicated that rebamipide and metronidazole exhibited preventive effects against an NSAID-induced enteropathy by promoting mucus production and shifting enteric bacteria, respectively [11,12]. However, the protective effects of these drugs have not been consistently observed in every study.

5-Aminosalicylic acid (5-ASA) and sulfasalazine, a prodrug of sulfapyridine and 5-ASA, have been frequently used for treating patients with inflammatory bowel diseases. Although the precise mechanisms for sulfasalazine and 5-ASA remain unknown, one plausible mechanism through which these drugs treat inflammatory bowel diseases is their free radical scavenging property [13]. Given that sulfasalazine and 5-ASA reduce intestinal inflammation and blood loss, they may be considered possible treatment options for an NSAID-induced enteropathy [14,15,16]. However, the protective effects of 5-ASA in the presence of NSAIDs have yet to be elucidated in small intestinal epithelial cells. Therefore, the current study investigated the molecular mechanisms through which 5-ASA improved an NSAID-induced enteropathy in small intestinal epithelial cells.

## 2. Materials and Methods

### 2.1. Cell Culture

An IEC-6 cell is a rat small intestinal cell line and remains a non-transformed, undifferentiated cell line [17]. IEC-6 cells were obtained from the Korean Cell Line Bank and cultured in Dulbecco’s Modified Eagle’s Medium with 4.5 g/L glucose and 2 mM glutamine supplemented with 10% fetal bovine serum, 1.5 g/L sodium bicarbonate, 100 µg/mL streptomycin and 100 IU/mL penicillin. The cells were maintained at 37 °C in 5% CO_2_ (Gibco, Grand Island, NY, USA).

### 2.2. 3-(4,5-Dimethylthiazol-2-yl)-2,5-Diphenyltetrazolium Dromide (MTT) Assay

An MTT (Sigma Chemicals, St. Louis, MO, USA) assay was used to measure cell proliferation as previously described [18]. Accordingly, cells were seeded onto a 96-well plate at a density of 5 × 10^4^ cells/mL. After incubation for 24 h, cells were treated with various concentrations of indomethacin and 5-ASA (Calbiochem, Gibbstown, NJ, USA) in a serum-free medium for 24 h. Each assay was performed in triplicate.

### 2.3. Cell Apoptosis Assay

Cell apoptosis was assessed by detecting mono-oligonucleosomes (histone-associated DNA fragments) using the cell death detection enzyme-linked immunosorbent assay (ELISA) plus kit (Roche Applied Science, Mannheim, Germany) according to the manufacturer’s instructions as previously described [18]. Accordingly, cells were seeded onto a 96-well plate at a density of 1 × 10^4^ cells/well and incubated for 24 h. Cells were then treated with various concentrations of indomethacin and 5-ASA for 48 h.

### 2.4. Caspase-3 Activity Assay

A caspase-3 activity assay kit (BioVision, Mountain View, CA, USA) was used to measure caspase-3 activity according to the manufacturer’s instructions as previously described [18]. Accordingly, cells were plated on 60 mm dishes at a density of 2 × 10^6^ cells/mL and treated with various concentrations of indomethacin and 5-ASA for 48 h.

### 2.5. Reactive Oxygen Species Measurement

Intracellular reactive oxygen species (ROS) levels were determined using a 2′,7′-dichlorofluorescein diacetate (DCFDA) Cellular ROS Detection Assay Kit (Abcam, Cambridge, UK) according to the manufacturer’s instructions. Briefly, IEC-6 cells were washed with Dulbecco’s phosphate-buffered saline and incubated with a 25 µM DCFDA mix for 45 min at 37 °C in the dark. Cells were then washed once with a buffer solution before drug treatment (indomethacin and 5-ASA) for 24 h. Signals were then detected every 5 min using a DTX 880 Multimode Detector (Beckman Coulter, Brea, CA, USA) at an excitation wavelength of 485 nm and an emission of 535 nm. Columns of non-stained cells were reserved as a blank control.

### 2.6. Superoxide Dismutase 2 (SOD2) Activity Measurement

Mitochondrial compartments were separated using a Mitochondria/Cytosol Fractionation kit (Bio Vision, Mountain View, CA, USA) as previously described [19]. This sample was used for the SOD2 activity assay (Bio Vision, Mountain View, CA, USA) at 450 nm using a microplate Epoch™ (BioTek, Winooski, VT, USA).

### 2.7. Western Blotting

Western blotting was performed as previously described [18]. Antibodies targeting occludin or zonula occludens-1 (ZO-1) were from Santa Cruz Biotechnology (Santa Cruz, CA, USA).

### 2.8. Immunofluorescence Staining

For immunofluorescence staining, IEC-6 cells were cultured on glass coverslips in 12-well plates and treated with indomethacin (100 µM) or 5-ASA (50 and 100 mM) for 24 h. Cells were then fixed in ice-cold methanol for 10 min and incubated with an occludin antibody (1:100) or a ZO-1 antibody (1:100) (Santa Cruz Biotechnology, United States) as previously described [20]. Cells on coverslips were incubated with DAPI (4′,6-diamidino-2-phenylindole) (1.5 µg/mL) (Vector Laboratories, St. Burlingame, CA, USA) for 1 min, after which images were obtained using a NiKonA1R (NIKON, Tokyo, Japan) and processed using NIS-Elements viewer 4.20 software (NIKON, Tokyo, Japan).

### 2.9. Wound Healing Assay

A wound healing assay was performed using IEC-6 cells incubated with indomethacin and 5-ASA either alone or in combination as previously described [18]. Experiments were carried out at least in triplicate.

## 3. Results

### 3.1. 5-Aminosalicylic Acid Restores Indomethacin-Induced Suppression of Cell Proliferation

**Figure 1:**
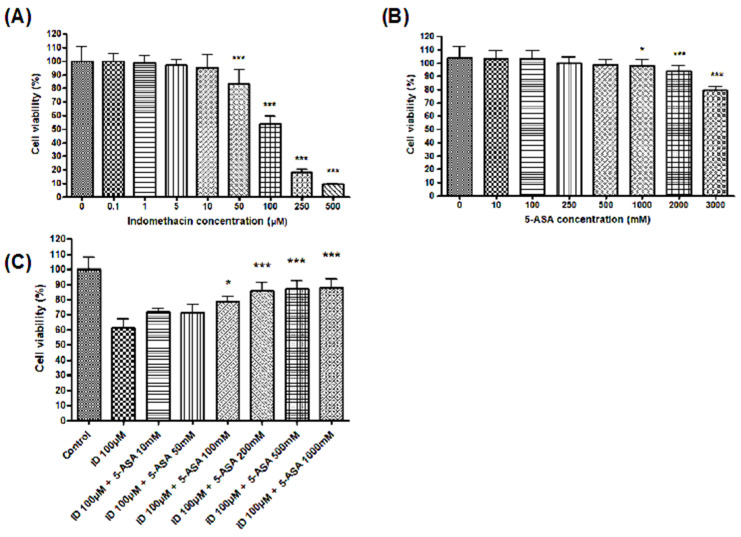
Effect of indomethacin and 5-ASA on cell proliferation. (**A**) Cell viability following treatment with various concentrations of indomethacin in IEC-6 cells determined using the 3-(4,5-Dimethylthiazol-2-yl)-2,5-Diphenyltetrazolium Dromide (MTT) assay. The indomethacin treatment dose dependently reduced cell viability. (**B**) Cell viability following treatment with various concentrations of 5-ASA in IEC-6 cells. 5-ASA treatment up to 500 mM did not reduce cell viability. (**C**) Cell viability following indomethacin and 5-ASA treatment. The 5-ASA dose dependently improved cell viability, which was suppressed by indomethacin. * *p* < 0.05, *** *p* < 0.001 vs. control. ID, indomethacin; 5-ASA, 5-aminosalicylic acid.

We performed MTT assays to measure the suppressive effects of indomethacin and 5-ASA on cell proliferation. The indomethacin dose dependently suppressed the IEC-6 cell proliferation (Figure 1A). Although 50 µM of indomethacin significantly reduced the cell proliferation activity, IEC-6 cells treated with up to 500 mM of 5-ASA showed no such decrease (Figure 1B). Accordingly, the 5-ASA dose dependently prevented the indomethacin-induced suppression of the cell proliferation (Figure 1C).

### 3.2. 5-Aminosalicylic Acid Ameliorates Indomethacin-Induced Cell Apoptosis

**Figure 2:**
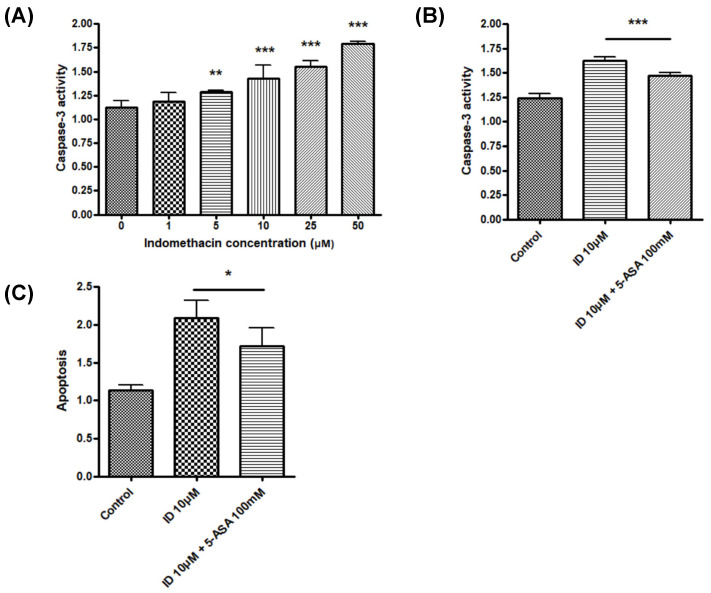
Effect of indomethacin and 5-ASA on cell apoptosis. (**A**) Caspase-3 activity following treatment with various concentrations of indomethacin for 48 h in IEC-6 cells. ** *p* < 0.01, *** *p* < 0.001 vs. control. (**B**) Caspase-3 activity following indomethacin and 5-ASA treatment in IEC-6 cells. *** *p* < 0.001. (**C**) Effect of indomethacin and 5-ASA on IEC-6 cell death determined using a cell death detection enzyme-linked immunosorbent assay. * *p* < 0.05. ID, indomethacin; 5-ASA, 5-aminosalicylic acid.

To determine the mode of cell death in the MTT assays, caspase-3 activity following indomethacin treatment was measured. Although indomethacin induced apoptosis by increasing caspase-3 activity in IEC-6 cells (Figure 2A), indomethacin-induced cell apoptosis was significantly ameliorated by 5-ASA (Figure 2B). The ELISA assay also revealed the same trend (Figure 2C).

### 3.3. 5-Aminosalicylic Acid Reduces Intracellular Reactive Oxygen Species and Induces Reactive Oxygen Species Scavenging

**Figure 3:**
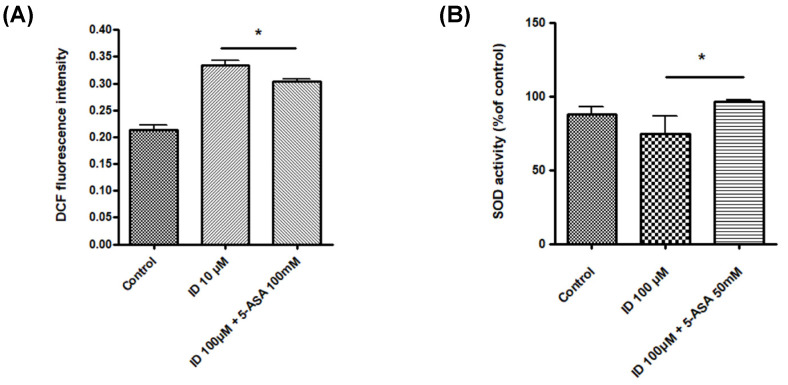
Effect of indomethacin and 5-ASA on intracellular reactive oxygen species (ROS) and scavenging. (**A**) Intracellular ROS contents following indomethacin and 5-ASA treatment in IEC-6 cells. (**B**) ROS scavenging activity following indomethacin and 5-ASA treatment in IEC-6 cells. * *p* < 0.05. ID, indomethacin; 5-ASA, 5-aminosalicylic acid.

Possible intracellular mechanisms through which indomethacin-induced IEC-6 cell apoptosis was investigated by measuring intracellular ROS contents. Although IEC-6 cells treated with indomethacin exhibited increased intracellular ROS contents (Figure 3A), 5-ASA was able to significantly reduce such an increase. Further investigation on SOD2 activity, which cleared mitochondrial ROS [16], showed that SOD2 activity was suppressed by indomethacin but augmented by 5-ASA in IEC-6 cells (Figure 3B).

### 3.4. 5-Aminosalicylic Acid Reversed Indomethacin-Induced Attenuation of Occludin and ZO-1 Expression

**Figure 4:**
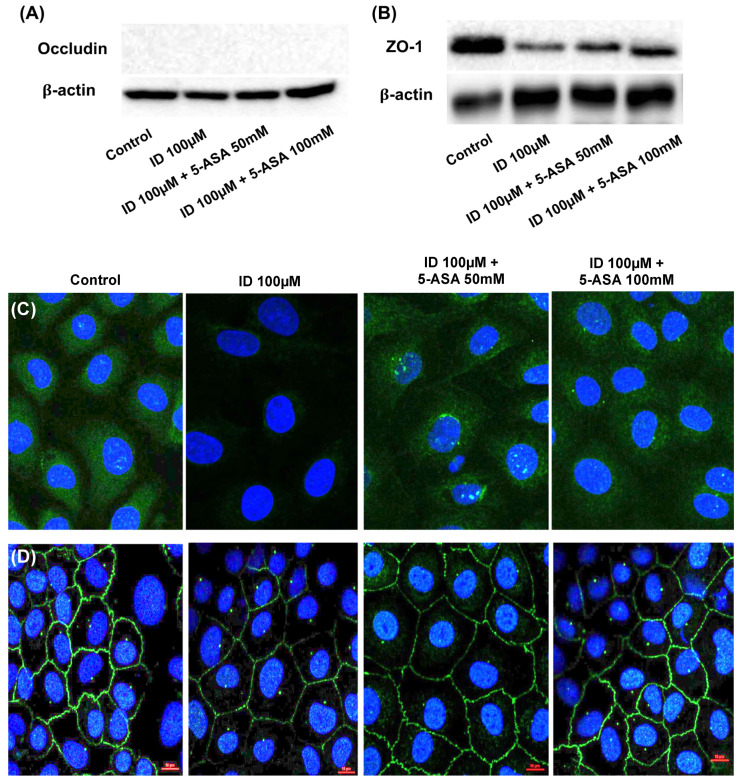
Effect of indomethacin and 5-ASA on tight junction molecule expressions in IEC-6 cells. Western blotting of occludin (**A**) and ZO-1 (**B**) following indomethacin and 5-ASA treatment. Immunofluorescence staining of occludin (**C**) and ZO-1 (**D)** following indomethacin and 5-ASA treatment (×100). ID, indomethacin; 5-ASA, 5-aminosalicylic acid.

To evaluate the effects of indomethacin and 5-ASA on epithelial permeability, the expression of two tight junction molecules, occludin and ZO-1, was measured after indomethacin treatment with or without 5-ASA. Western blotting of occludin and ZO-1 revealed that indomethacin reduced the expression of the aforementioned tight junction molecules whereas 5-ASA attenuated it (Figure 4A,B, respectively). Immunofluorescence staining also suggested that 5-ASA attenuated the indomethacin-induced reduction in the expression of occludin and ZO-1 (Figure 4C,D, respectively).

### 3.5. 5-Aminosalycilic Acid Promotes Wound Healing

**Figure 5:**
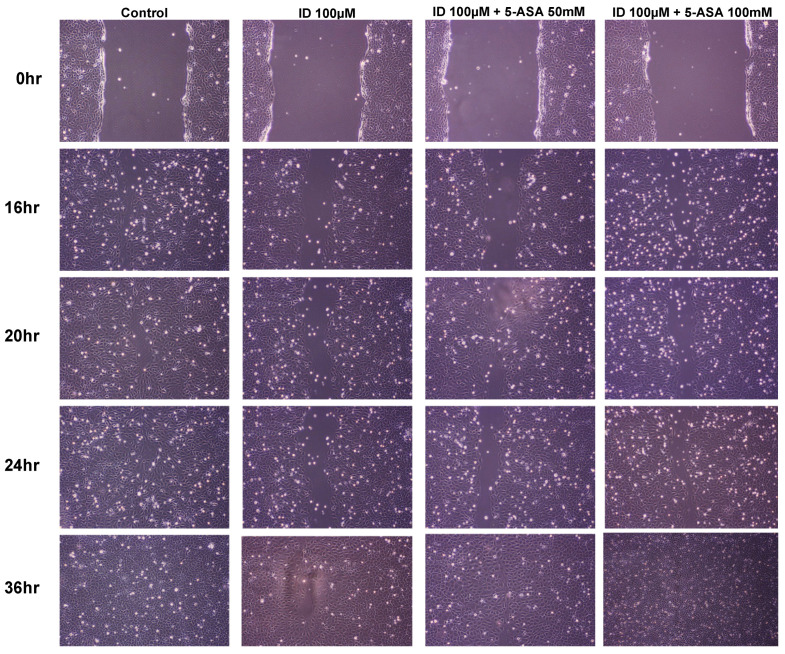
Effect of indomethacin and 5-aminosalicylate on wound healing. Cell migration or protrusion was photographed at 0, 16, 20, 24 and 36 h after wounding IEC-6 cells (×200). ID, indomethacin; 5-ASA, 5-aminosalicylic acid.

To determine the effects of 5-ASA on wound healing, a wound healing assay was performed. Accordingly, IEC-6 cells treated with indomethacin had slower wound healing compared with the control. IEC-6 cells cotreated with indomethacin and 5-ASA exhibited faster wound healing than cells treated with indomethacin alone in a dose dependent manner (Figure 5).

## 4. Discussion

The current study confirmed that indomethacin suppressed the proliferation and induced apoptosis of IEC-6 cells, both of which were ameliorated by the coadministration of 5-ASA. Our results suggest that 5-ASA reduced indomethacin toxicity by scavenging intracellular ROS. Studies have suggested that NSAIDs cause small bowel damage through cyclooxygenase (COX)-dependent and -independent mechanisms [3]. In the COX-dependent mechanism, NSAIDs inhibit prostaglandin synthesis, which plays an important role in the mucosal barrier function, leading to increased mucosal permeability. Plasma proteins subsequently leak into the lumen, causing bacterial overgrowth followed by neutrophil infiltration into mucosa and intestinal ulceration. However, small bowel injury is considered to be less dependent on COX-1 inhibition than gastric injury [21]. The COX-independent mechanism can be explained by the three-hit hypothesis. The first step involves NSAIDs directly damaging surface membrane phospholipids, which results in mucosal damage and enterocyte mitochondrial damage. The second step involves mitochondrial damage leading to calcium efflux and free radical generation, which cause a breakdown in intercellular integrity and subsequent increase in intestinal permeability. The third and final step involves the loss of the mucosal barrier, allowing mucosal damage by intraluminal contents such as bile, protease, toxin and bacteria. Accordingly, the indomethacin-induced increase in intracellular ROS and the suppression of SOD2 activity in IEC-6 cells are related to the COX-independent mechanism for NSAID-induced small bowel injuries.

On the other hand, our results revealed that 5-ASA cotreatment reduced intracellular ROS and restored SOD2 activity. This may have been attributed to its role as a free radical scavenger and antioxidant within the intestinal mucosa, one of the previously known mechanisms by which 5-ASA exhibits anti-inflammatory effects [13,22,23]. In vitro experiments have determined that 5-ASA inhibits proinflammatory cytokines by inducing a PPAR-γ gene expression [24], functions as a free radical scavenger [13] and exhibits immunosuppressive activity [25]. Moreover, Burress et al. reported that 5-ASA exhibited antioxidant effects by inducing an intestinal epithelial heat shock protein (hsp72) expression [26]. Overall, the theory of an NSAID-induced enteropathy suggests that the ROS scavenging activity of 5-ASA allows it to inhibit apoptosis and promote cell proliferation, thereby protecting against an NSAID-induced small intestinal injury.

We further investigated the effects of 5-ASA on intestinal permeability. Accordingly, Western blotting and immunofluorescence staining showed that while indomethacin inhibited occludin and ZO-1 expression in IEC-6 cells, 5-ASA cotreatment restored their expression. 5-ASA cotreatment also promoted wound healing, which was reduced by indomethacin. Taken together, the aforementioned results suggest that 5-ASA could reduce intestinal permeability by restoring tight junction molecule expression and promoting wound healing.

5-ASA has been an emerging treatment option for managing NSAID-induced enteropathies based on experience with their application in inflammatory bowel diseases, particularly ulcerative colitis [14,15,16]. Moreover, one in vivo experiment using rat models showed that mesalazine reversed the morphological and functional changes induced by indomethacin [27]. Furthermore, Nandi et al. reported that the 5-ASA-induced inhibition of the inducible isoform of NO synthase (iNOS) improved intestinal ulceration in an indomethacin-induced enteropathy [28]. To the best of our knowledge, this has been the first study to elucidate the cellular mechanisms of 5-ASA in an NSAID-induced small intestinal injury.

## 5. Conclusions

The current study revealed that 5-ASA ameliorated an NSAID-induced small intestinal cell injury by promoting cell proliferation, inhibiting apoptosis and inducing the expression of tight junction proteins, all of which could be attributed to its free radical scavenging function. Therefore, the present study provides evidence that 5-ASA could be a potential treatment option for an NSAID-induced enteropathy. More preclinical and clinical studies are needed to confirm the effectiveness of 5-ASA on NSAID-induced enteropathies.

## References

[1] Kasciuskeviciute S., Gumbrevicius G., Vendzelyte A., Sciupokas A., Petrikonis K., Kadusevicius E. (2018). Impact of the World Health Organization Pain Treatment Guidelines and the European Medicines Agency Safety Recommendations on Nonsteroidal Anti-Inflammatory Drug Use in Lithuania: An Observational Study. Medicina.

[2] Allison M.C., Howatson A.G., Torrance C.J., Lee F.D., Russell R.I. (1992). Gastrointestinal damage associated with the use of nonsteroidal antiinflammatory drugs. N. Engl. J. Med..

[3] Fortun P.J., Hawkey C.J. (2005). Nonsteroidal antiinflammatory drugs and the small intestine. Curr. Opin. Gastroenterol..

[4] Goldstein J.L., Eisen G.M., Lewis B., Gralnek I.M., Zlotnick S., Fort J.G., Investigators (2005). Video capsule endoscopy to prospectively assess small bowel injury with celecoxib, naproxen plus omeprazole, and placebo. Clin. Gastroenterol. Hepatol..

[5] Graham D.Y., Opekun A.R., Willingham F.F., Qureshi W.A. (2005). Visible small-intestinal mucosal injury in chronic NSAID users. Clin. Gastroenterol. Hepatol..

[6] Maiden L., Thjodleifsson B., Seigal A., Bjarnason I.I., Scott D., Birgisson S., Bjarnason I. (2007). Long-term effects of nonsteroidal anti-inflammatory drugs and cyclooxygenase-2 selective agents on the small bowel: A cross-sectional capsule enteroscopy study. Clin. Gastroenterol. Hepatol..

[7] Maiden L., Thjodleifsson B., Theodors A., Gonzalez J., Bjarnason I. (2005). A quantitative analysis of NSAID-induced small bowel pathology by capsule enteroscopy. Gastroenterology.

[8] Pennazio M., Rondonotti E., Pellicano R., Cortegoso Valdivia P. (2020). Small bowel capsule endoscopy: Where do we stand after 20 years of clinical use?. Minerva Gastroenterol. Dietol..

[9] Wallace J.L., Syer S., Denou E., de Palma G., Vong L., McKnight W., Jury J., Bolla M., Bercik P., Collins S.M. (2011). Proton pump inhibitors exacerbate NSAID-induced small intestinal injury by inducing dysbiosis. Gastroenterology.

[10] Watanabe T., Sugimori S., Kameda N., Machida H., Okazaki H., Tanigawa T., Watanabe K., Tominaga K., Fujiwara Y., Oshitani N. (2008). Small bowel injury by low-dose enteric-coated aspirin and treatment with misoprostol: A pilot study. Clin. Gastroenterol. Hepatol..

[11] Niwa Y., Nakamura M., Ohmiya N., Maeda O., Ando T., Itoh A., Hirooka Y., Goto H. (2008). Efficacy of rebamipide for diclofenac-induced small-intestinal mucosal injuries in healthy subjects: A prospective, randomized, double-blinded, placebo-controlled, cross-over study. J. Gastroenterol..

[12] Bjarnason I., Hayllar J., Smethurst P., Price A., Gumpel M.J. (1992). Metronidazole reduces intestinal inflammation and blood loss in non-steroidal anti-inflammatory drug induced enteropathy. Gut.

[13] Ahnfelt-Ronne I., Nielsen O.H., Christensen A., Langholz E., Binder V., Riis P. (1990). Clinical evidence supporting the radical scavenger mechanism of 5-aminosalicylic acid. Gastroenterology.

[14] Hayllar J., Smith T., Macpherson A., Price A.B., Gumpel M., Bjarnason I. (1994). Nonsteroidal antiinflammatory drug-induced small intestinal inflammation and blood loss. Effects of sulfasalazine and other disease-modifying antirheumatic drugs. Arthritis Rheum..

[15] Bjarnason I., Hopkinson N., Zanelli G., Prouse P., Smethurst P., Gumpel J.M., Levi A.J. (1990). Treatment of non-steroidal anti-inflammatory drug induced enteropathy. Gut.

[16] Racz I., Szalai M., Kovacs V., Regoczi H., Kiss G., Horvath Z. (2013). Mucosal healing effect of mesalazine granules in naproxen-induced small bowel enteropathy. World J. Gastroenterol..

[17] Quaroni A., Wands J., Trelstad R.L., Isselbacher K.J. (1979). Epithelioid cell cultures from rat small intestine. Characterization by morphologic and immunologic criteria. J. Cell Biol..

[18] Lee J., Hong E.M., Kim J.H., Jung J.H., Park S.W., Koh D.H., Choi M.H., Jang H.J., Kae S.H. (2019). Metformin Induces Apoptosis and Inhibits Proliferation through the AMP-Activated Protein Kinase and Insulin-like Growth Factor 1 Receptor Pathways in the Bile Duct Cancer Cells. J. Cancer.

[19] Omatsu T., Naito Y., Handa O., Mizushima K., Hayashi N., Qin Y., Harusato A., Hirata I., Kishimoto E., Okada H. (2010). Reactive oxygen species-quenching and anti-apoptotic effect of polaprezinc on indomethacin-induced small intestinal epithelial cell injury. J. Gastroenterol..

[20] Jang H.J., Hong E.M., Kim M., Kim J.H., Jang J., Park S.W., Byun H.W., Koh D.H., Choi M.H., Kae S.H. (2016). Simvastatin induces heme oxygenase-1 via NF-E2-related factor 2 (Nrf2) activation through ERK and PI3K/Akt pathway in colon cancer. Oncotarget.

[21] Wallace J.L. (1997). Nonsteroidal anti-inflammatory drugs and gastroenteropathy: The second hundred years. Gastroenterology.

[22] Craven P.A., Pfanstiel J., Saito R., DeRubertis F.R. (1987). Actions of sulfasalazine and 5-aminosalicylic acid as reactive oxygen scavengers in the suppression of bile acid-induced increases in colonic epithelial cell loss and proliferative activity. Gastroenterology.

[23] Grisham M.B., Granger D.N. (1989). 5-Aminosalicylic acid concentration in mucosal interstitium of cat small and large intestine. Dig. Dis. Sci..

[24] Rousseaux C., Lefebvre B., Dubuquoy L., Lefebvre P., Romano O., Auwerx J., Metzger D., Wahli W., Desvergne B., Naccari G.C. (2005). Intestinal antiinflammatory effect of 5-aminosalicylic acid is dependent on peroxisome proliferator-activated receptor-gamma. J. Exp. Med..

[25] Stevens C., Lipman M., Fabry S., Moscovitch-Lopatin M., Almawi W., Keresztes S., Peppercorn M.A., Strom T.B. (1995). 5-Aminosalicylic acid abrogates T-cell proliferation by blocking interleukin-2 production in peripheral blood mononuclear cells. J. Pharmacol. Exp. Ther..

[26] Burress G.C., Musch M.W., Jurivich D.A., Welk J., Chang E.B. (1997). Effects of mesalamine on the hsp72 stress response in rat IEC-18 intestinal epithelial cells. Gastroenterology.

[27] Simon H., Fischer T., Almasi A., Fischer E. (2017). Effects of Mesalazine on Morphological and Functional Changes in the Indomethacin-Induced Inflammatory Bowel Disease (Rat Model of Crohn’s Disease). Pathol. Oncol. Res..

[28] Nandi J., Saud B., Zinkievich J.M., Palma D.T., Levine R.A. (2008). 5-aminosalicylic acid improves indomethacin-induced enteropathy by inhibiting iNOS transcription in rats. Dig. Dis. Sci..

